# Expansion of Intestinal Epithelial Stem Cells during Murine Development

**DOI:** 10.1371/journal.pone.0027070

**Published:** 2011-11-10

**Authors:** Jeffrey J. Dehmer, Aaron P. Garrison, Karen E. Speck, Christopher M. Dekaney, Laurianne Van Landeghem, Xiaofei Sun, Susan J. Henning, Michael A. Helmrath

**Affiliations:** 1 Department of Surgery, University of North Carolina, Chapel Hill, North Carolina, United States of America; 2 Department of Cell and Molecular Physiology, University of North Carolina, Chapel Hill, North Carolina, United States of America; 3 Department of Surgery, Cincinnati Children's Hospital Medical Center, Cincinnati, Ohio, United States of America; 4 Perinatal Institute, Cincinnati Children's Hospital Medical Center, Cincinnati, Ohio, United States of America; 5 Department of Medicine, University of North Carolina, Chapel Hill, North Carolina, United States of America; Brigham & Women's Hospital - Harvard Medical School, United States of America

## Abstract

Murine small intestinal crypt development is initiated during the first postnatal week. Soon after formation, overall increases in the number of crypts occurs through a bifurcating process called crypt fission, which is believed to be driven by developmental increases in the number of intestinal stem cells (ISCs). Recent evidence suggests that a heterogeneous population of ISCs exists within the adult intestine. Actively cycling ISCs are labeled by *Lgr5*, *Ascl2* and *Olfm4*; whereas slowly cycling or quiescent ISC are marked by *Bmi1* and *mTert*. The goal of this study was to correlate the expression of these markers with indirect measures of ISC expansion during development, including quantification of crypt fission and side population (SP) sorting. Significant changes were observed in the percent of crypt fission and SP cells consistent with ISC expansion between postnatal day 14 and 21. Quantitative real-time polymerase chain reaction (RT-PCR) for the various ISC marker mRNAs demonstrated divergent patterns of expression. *mTert* surged earliest, during the first week of life as crypts are initially being formed, whereas *Lgr5* and *Bmi1* peaked on day 14. *Olfm4* and *Ascl2* had variable expression patterns. To assess the number and location of *Lgr5*-expressing cells during this period, histologic sections from intestines of *Lgr5*-EGFP mice were subjected to quantitative analysis. There was attenuated *Lgr5*-EGFP expression at birth and through the first week of life. Once crypts were formed, the overall number and percent of *Lgr5*-EGFP positive cells per crypt remain stable throughout development and into adulthood. These data were supported by Lgr5 *in situ* hybridization in wild-type mice. We conclude that heterogeneous populations of ISCs are expanding as measured by SP sorting and mRNA expression at distinct developmental time points.

## Introduction

The structure and physiology of the intestinal tract mature during postnatal growth in order to meet changing absorptive and digestive requirements. While the mechanisms and timing of certain critical events differ between species, the maturation of bowel structure and function is universally important. In rodents, the postnatal growth of the epithelium has been studied in considerable detail [1], [2]. Central to the increase in both intestinal length and circumference is the observation that the number and depth of crypts increases significantly between the second and fourth weeks of postnatal life [1], [2]. A major determinant of this increase in crypt number is crypt fission (also called crypt branching or crypt bifurcation).

Prior work has characterized crypt fission in various mammalian species during the postnatal time period [1], [3]–[5]. Cheng and Bjerknes described a peak of crypt fission at 2 weeks of age in mice [1]. Several groups have characterized the process in rats, with fission peaking during postnatal weeks 2–3 [4], [5]. In human infants, the rate of crypt fission was highest between 6–12 months of age as reported by Cummins' group [3]. All of these data indicate that crypt fission is a critical process in intestinal development. Although the proximate causes of crypt fission are unclear and may be due to mesenchymal, luminal or hormonal influences, an increase in crypt volume has been proposed as a trigger for this process. Specifically, Totafurno et al. developed a crypt growth model which demonstrates that crypts will bifurcate when their volume doubles [6]. Inherent in this model is the prediction that a doubling in the number of stem cells per crypt is the driving force for crypt fission. Despite this prediction having been made more than 20 years ago, there are no reported studies that have directly investigated the behavior of pluripotent intestinal stem cells (ISCs) during the postnatal period when extensive crypt fission is occurring. An in depth review of epithelial stem cells by Wright further highlights the crucial relationship between crypt fission and stem cell number [7].

Recent developments in the characterization of ISCs have led to the identification of multiple putative ISC markers. To date however, there is no consensus regarding the ideal marker and no single one appears to universally identify these important pluripotent cells [8]. Furthermore, there is ongoing debate about the true location of the ISC. Lineage tracing and gene signature work indicates that columnar cells located in the crypt base amongst the Paneth cells possess many “stem-like” characteristics, namely generation of all differentiated intestinal cell lineages and self-renewal [9], [10]. Alternatively, a position above the Paneth cells has been proposed based on other data, including label retention studies [11], [12].

Various lines of evidence suggest that the intra-Paneth cell ISC (crypt base cells or CBCs) and the supra-Paneth cell ISC (often referred to as “plus 4”) represent distinct sub-populations that may be active and quiescent, respectively [10], [13], [14]. In light of these issues and controversies, we selected several putative ISC markers which can be used to identify both groups of ISC [15]–[21]. Comprising the presumed “active” ISCs, *Lgr5*, *Ascl2* and *Olfm4* are markers of the crypt base columnar cell population [15], [20], [21], while *Bmi1* and *mTert* identify the supra-Paneth “quiescent” ISCs [16]–[19], [22]. Our goal was to characterize the behavior of ISCs within the time frame of postnatal murine development, when significant intestinal growth occurs. We hypothesized that expansion of ISCs would be temporally related to increases in crypt fission.

## Methods

### Animals

All animal experiments were carried out after approval by the University of North Carolina School of Medicine Institutional Animal Care and Use Committee (protocol number 07-202.0). Adult male and female wild-type mice (WT, C57BL/6J) and breeder pair heterozygote *Lgr5*-EGFP-IRES-creERT2 (*Lgr5*-EGFP–enhanced green fluorescent protein) mice were obtained from The Jackson Laboratory (Bar Harbor, ME) and paired in order to obtain timed pregnancies. *Lgr5*-EGFP genotyping was performed on tail clippings using Extract-N-Amp Tissue PCR kits (Sigma-Aldrich, St. Louis, MO) with common (CTGCTCTCTGCTCCCAGTCT), WT reverse (ATACCCCATCCCTTTTGAGC) and mutant reverse primers (GAACTTCAGGGTCAGCTTGC) to perform the PCR as described by The Jackson Laboratory (http://jaxmice.jax.org/strain/008875.html). Offspring were euthanized on postnatal days (p) 0, 4, 7, 14, 21, 28 and adulthood (8–12 weeks) after induction of 2% isoflurane general anesthesia. Each data point is represented by 3 or more animals from both sexes with actual numbers given in the respective figure legends. All animals were weaned to standard chow (Prolab RMH 3000, LabDiet, St. Paul, MN) on p21 and kept under a 12 hour light-dark cycle.

### Tissue Harvest

The entire small bowel was eviscerated and its length recorded. The proximal 1 cm was discarded as duodenum and the remaining small bowel was divided in half with the proximal portion being the jejunum. The middle 2 cm of jejunum was isolated and flushed with phosphate-buffered saline. The first cm was placed in 10% buffered zinc formalin (Fisher Scientific, Kalamazoo, MI) for histology and the second 1 cm was cut in several smaller pieces, and then placed in FastPrep-24 Lysing Matrix D tubes (MP Biomedicals, Solon, OH) filled with 600 µL of RLT lysis buffer, a component of the Qiagen RNeasy Mini Kit (Qiagen, Valencia, CA). These samples were snap frozen in liquid nitrogen and kept at −80°C until RNA isolation. For *Lgr5*-EGFP mice, an adjacent 3rd cm was harvested and sequentially placed in 4% paraformaldehyde, 30% sucrose, and then OCT compound (Sakura Finetek, Torrance, CA) and stored at −80°C until slide preparation.

### Histology

For studies with WT mice, slides were stained with hematoxylin and eosin (H&E) and analyzed for intestinal morphometry in a blinded manner. Images were obtained using an AxioImager microscope (Zeiss, Thornwood, NY). Crypt depth and villus height were obtained from at least 10 well-oriented crypt-villus axes per animal. Circumference measurements were obtained from the submucosa using AxioVision 4.6 as described previously [23]. The number of fissioning crypts was determined by observing at least 100 crypts per animal for a bifurcation with a fissure creating 2 flask-shaped bases with a shared single crypt-villus junction. The number of crypts per circumference was counted in intact transverse sections.

For studies with *Lgr5*-EGFP mice, we utilized a laser scanning spectral confocal microscopy system and software (Leica TCS SP2, Bannockburn, IL) to visualize *Lgr5*-EGFP sections by fluorescence and take representative photographs. To quantitate the number of EGFP positive cells per crypt: a) only complete, well oriented crypts were chosen; b) only cells exhibiting bright fluorescence were counted; and c) within the focal range of the section all such cells (i.e. from both sides of the crypt) were counted. For the same crypts the total number of cells were quantitated by scoring the number of nuclei that stained positive for 4′,6-diamidino-2-phenylindole (DAPI). Preliminary analyses did not show gross differences in the absolute number of EGFP positive cells per crypt or the% EGFP positive cells/crypt from p7–p28, therefore animals were grouped to provide an n of 3–5 during early (p7–p14) and late (p21–p28) development for comparison with adulthood. For quantification of the percentage of EGFP positive crypts, at least 2 transverse sections were examined per animal.

### RNA isolation and quantitative real time PCR

Total RNA was isolated using the Qiagen RNeasy Mini Kit per the manufacturer's instructions after addition of 6 µL β-mercaptoethanol (Sigma-Aldrich, St. Louis, MO) to the lysing tubes. Complimentary DNA was then created using a High Capacity cDNA Reverse Transcription Kit (Applied Biosystems, Foster City, CA). Quantitative RT-PCR was performed on cDNA samples in triplicate using an Applied Biosystems StepOne Plus system. Individual probes for *Lgr5* (Mm00438890_m1), *Ascl2* (Mm01268891_g1), *Bmi1* (Mm03053308_g1), *Olfm4* (Mm01320260_m1), and *mTert* (Mm01352136_m1) from Applied Biosystems were used and data analyzed using a relative standard curve method normalized to *β-actin* (Mm00607939_s1). We evaluated *18S*, *GAPDH* and *β-actin* and found that the latter changed the least across our developmental time points and thus was used for the housekeeping gene.

### In situ hybridization

*In situ* hybridization was performed on WT animals as previously described [24]. In brief, 12 µm frozen sections were mounted onto poly-L-lysine coated slides and fixed in cold 4% (wt/vol) paraformaldehyde in phosphate buffered saline. The sections were prehybridized and hybridized at 45°C for 4 h in 50% (vol/vol) formamide hybridization buffer containing the ^35^S-labeled antisense or sense cRNA probes. These were prepared using nucleotides 2538 to 3268 (NCBI reference sequence NM_010195.2) of *Lgr5* cDNA cloned into a pBluescript vector. RNase A-resistant hybrids were detected by autoradiography. Sections were post-stained with H&E. Sections hybridized with the sense probes did not exhibit any positive signals and served as negative controls as shown in Figure S2.

### Side population sorting

Jejunal mucosal cell suspensions were prepared from p21 and adult mice as previously described using a collagenase/dispase method [23]. Following staining with Hoechst 33342 and FITC-labeled anti-CD45 as described by Dekaney et al. [25], the percent of CD45^−^ SP cells was quantitated using flow cytometry.

### Statistics

Data are presented as means ± standard error. All timepoints and ranges are represented by an n ≥3 animals. Statistics were performed using SAS version 9.2 (SAS Institute, Cary, NC). To analyze the qRT-PCR results, a regression model was fit with each stem cell marker as the response and dummy variables for the timepoints as predictors. Levene's test for homeogeneity of variance was performed and if it showed heterogeneity then a weighted-least-squares model was fit with weights inversely proportional to the variance of the response at that time. For each marker, a test of any overall difference between times was done and if statistically significant, pairwise tests were performed. P-values less that 0.05 were considered significant. A full table of p-values for the analysis of qRT-PCR results and crypt fission is shown in Table S1. EGFP data was analyzed by 1-way analysis of variance followed by post-hoc Student's t-tests. Side population data was analyzed using Student's t-test.

## Results

**Figure 1:**
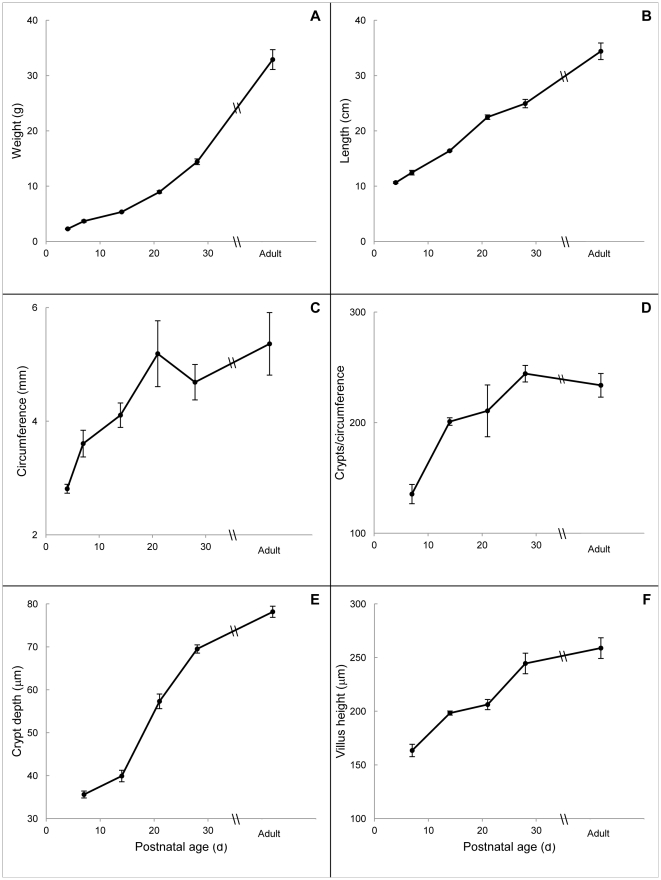
Changes in intestinal morphology of WT mice during development. For all panels, the x-axis represents postnatal age in days. Panel A shows body weight and panel B shows total small bowel length. All remaining panels show data from the mid-jejunum. As crypts are not well formed by p4, panels D–F show results from p7 onward. Data are given as means ± standard error (n = 3–17).

Over the first 6 weeks of intestinal development, morphologic parameters increased in a gradual fashion as expected (Fig. 1, panels A–F). Animal weight, small intestinal length, crypt depth and villus height increased in a linear fashion with R^2^ values of 0.91, 0.99, 0.94 and 0.92, respectively (Fig. 1 A,B,E,F). In contrast, jejunal circumference and the number of crypts per circumference increased during the first 2 postnatal weeks then plateaued from p21 onward, thus showing a more logarithmic pattern with R^2^ values of 0.92 and 0.96 (Fig. 1C and D).

**Figure 2:**
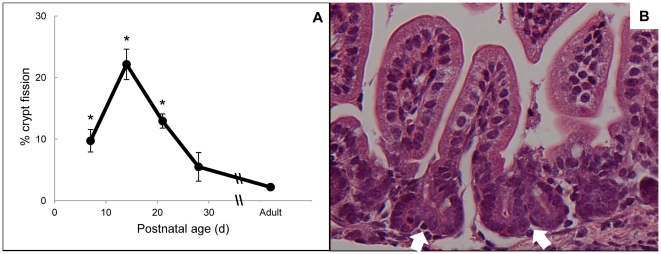
Changes in jejunal crypt fission during development of WT mice. Data in Panel A are given as means ± SE (n = 3–6). Panel B shows H&E stained jejunum from a p14 mouse and demonstrates examples of crypt fission (white arrows) at 40X magnification. The scale bar in Panel B is 20 µm. * indicates p<0.01 compared with adult.

As seen in Fig. 2A, crypt fission was observed at relatively high levels throughout the first 3 postnatal weeks, with a peak at p14, at which point 22.1±2.5% of jejunal crypts were scored as fissioning. The percentage of fissioning crypts remained significantly elevated at p21 then declined by p28 to levels that were not significantly different from adulthood. These baseline levels of fission are consistent with reports of crypt fission in untreated adult mice [1]. A representative photomicrograph of an animal at p14 is shown in Fig. 2B, with black arrows demonstrating fissioning crypts.

**Figure 3:**
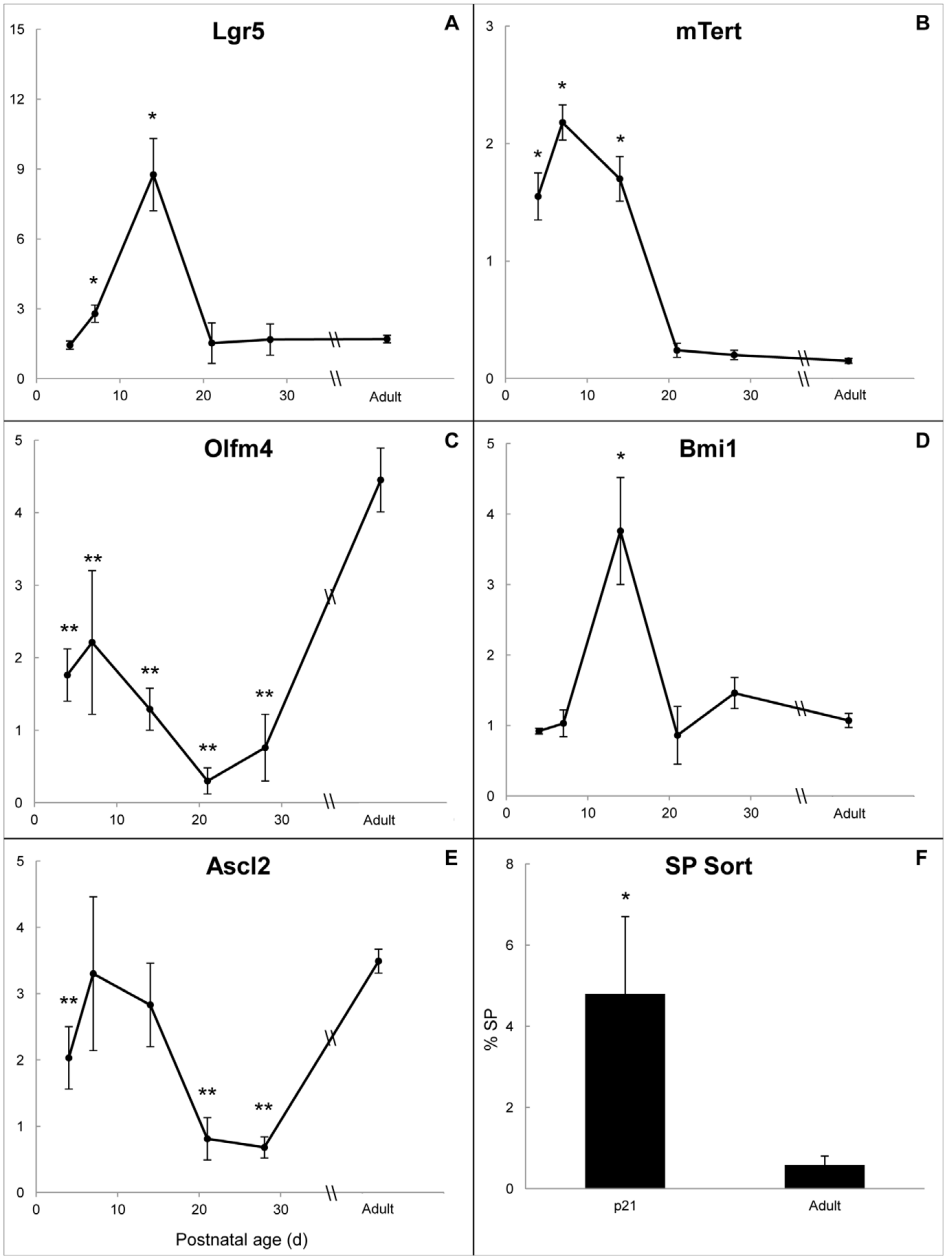
ISC marker mRNA expression in WT mice and percent SP cells at p21 and adulthood. For panels A–E, the x-axis represents postnatal age in days and the y-axis represents fold change in mRNA expression compared with β-actin. Markers for the CBC subpopulation are shown in the left (panels A, C, and E), whereas markers for the “plus 4” ISC subpopulation are shown on the right (panels B and D). Panel F shows the percentage of CD45^−^ side population (%SP) from p21 and adult animals. For all panels, data are given as means ± SE (n = 3–6) and * indicates values significantly higher than adult (p<0.01) whereas ** indicates values significantly lower than adult (p<0.01). Data for the entire statistical analysis of the mRNA data are shown in Supplemental Table 1.

In order to explore the hypothesis that developmental crypt fission is driven by increased numbers of ISCs, our first approach was to perform quantitative RT-PCR for several putative mRNA markers of ISC as shown in Fig. 3 (panels A–E). Of these, *mTert* mRNA (Fig. 3B) surged earliest, at p4, and remained elevated at p7 and p14, then declined to adult levels by p21. *Lgr5* and *Bmi1* mRNA expression peaked at p14 (Figs. 3A and 3D), corresponding with the peak of crypt fission. *Ascl2* mRNA (Fig 3E) was not significantly different from adult at these time points. Additionally, *Olfm4* mRNA (Fig 3C) remained significantly lower than adulthood at all neonatal time points.

In addition to crypt fission, we utilized another indirect method to confirm ISC expansion during this time period. SP sorting has been used previously to isolate and quantify an ISC enriched fraction from the epithelium [23], [25], [26]. As crypt fission was still elevated at p21 in our model, we compared this time point to adult animals. Our results (Fig. 3F) indicated that the% SP was significantly elevated at p21 compared with adult. Ages younger than p21 were not studied in this experiment because the fragility of the tissue precluded epithelial preparations comparable with those from adult animals.

**Figure 4:**
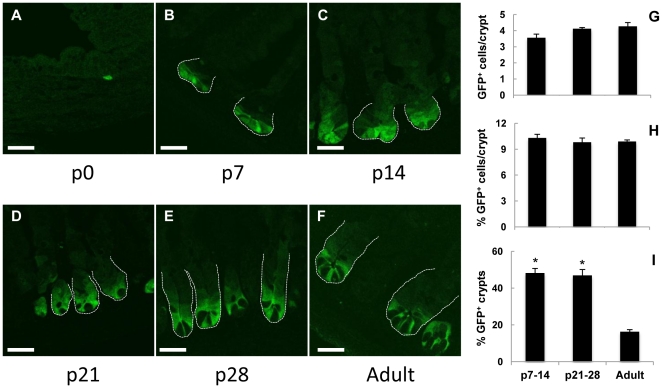
*Lgr5*-EGFP cells from neonatal and adult intestinal crypts. Panels A–F are representative confocal EGFP images of *Lgr5*-EGFP mouse jejunum from p0, 7, 14, 21, 28 and adult shown at 40X optical magnification with 2X digital zoom. The scale bar is 20 µm. Panel G shows the absolute number of EGFP positive cells per crypt and panel H the percent EGFP positive cells per crypt. Panel I shows the total percent of EGFP positive crypts in transverse sections. * indicates a significantly higher% of EGFP positive crypts in development compared with all adulthood (p<0.002).

To assess whether there are increased numbers of *Lgr5*-expressing ISCs during the period of elevated crypt fission (p7–p21), we utilized the commercially available *Lgr5*-EGFP mouse in order to visualize the *Lgr5*-expressing cells in the crypts. Representative confocal photomicrographs of *Lgr5*-EGFP animals are shown in Fig. 4 (panels A–F). At p0, EGFP positive cells were very sporadic in the intervillous region, with only 1–2 EGFP positive cells seen across all observed sections. By p7, a mosaic pattern of EGFP expression was apparent with approximately half the crypts being EGFP positive. In the positive crypts, EGFP localized only to the very base. Over time, as crypt depth increased (Fig. 1E), there was a trend toward more EGFP positive cells seen in each crypt (Fig. 4B–F). Starting at p21 (Fig. 4D), darker areas in the crypt base correspond with the appearance of Paneth cells and at the adult time point (Fig. 4F), fluorescence in the crypt base appeared restricted to several tall columnar cells located throughout the crypt base, consistent with the original report of these mice [15]. Quantitative data did not demonstrate more EGFP positive cells per crypt in development compared with adult (Fig. 4G). To the contrary there was a gradual trend towards increased numbers of EGFP cells per crypt, from 3.6±0.2 at p7–14 to 4.3±0.2 in adulthood (Fig. 4C) which parallels the increase in total number of cells per crypt, from 32.7±1.1 to 38.3±0.7 (data not shown). Consequently, the percentage of EGFP positive cells per crypt remained constant (Fig. 4H) as crypts increased in overall cellularity. The percentage of total crypts that were EGFP positive remained at approximately 50% from p7–p28, then decreased significantly, with only 17.3% ± 1.1 of crypts remaining EGFP positive in adulthood (Fig. 4I).

### Discussion

Mature bowel possesses an ability to respond to challenges by augmenting several characteristics after a loss of length or functional capacity [23], [26], [27]. This behavior is also evident during development, a time marked by significant increases in both macroscopic and microscopic features of the intestine. A careful analysis of the expansion of the murine intestinal epithelium during postnatal development has been described by Cheng and Bjerknes [1]. Although they utilized Swiss albino mice while we studied C57BL/6J animals, overall trends for common measures such as crypt depth and villus height were similar, as was the timing of the peak of crypt fission.

Two parameters included in our study but not reported in the previous study were bowel circumference and the number of crypts per circumference. Bowel circumference has particular clinical relevance as dilation is one of the properties of small intestinal adaptation after massive bowel resection both in mice and in human patients [23], [28]. Measuring the crypts per circumference offers additional insight into the rate of crypt formation and the relationship of crypt fission to the postnatal increase of bowel circumference. As can be seen in Figs. 1 and 2, the high rates of crypt fission at p7–21 are temporally associated with the increases in bowel circumference and the number of crypts per circumference. Then as crypt fission declines to low levels at p28 to adulthood, both of these circumference parameters plateau. These observations support the conclusion that during development, just as following resection and damage, crypt fission and the resulting increase in crypt number is imperative for improving functional capacity of the bowel [23], [26].

The process of crypt fission has been described in rodents and humans [1], [3], [4]. The most interesting aspect of this process is its temporal restriction. In each species, there is a peak of fission during development, followed by establishment of a baseline level in adults. In rats, fission peaks on p11 at 10.5% and remains elevated until p25, when it declines to adult baseline levels [4]. In humans, a peak is seen between 6–12 months of age at 18% [3]. Prior work in Swiss albino mice showed a peak in the number of bifid crypts (35%) at 2–3 weeks of life [1]. This corresponds to our current data demonstrating a peak of 22.1% on p14. Interestingly, both in rodents and humans, subsequent decline of crypt fission corresponds with the expected time of weaning (although specific data on weaning were not available from the human study). Certainly, this raises the possibility that factors delivered in the milk may be responsible, at least in part, for the high rates of crypt fission during the suckling period. Crypt fission is also temporally restricted in models of intestinal resection, where a loss of length stimulates ISCs to proliferate and generate additional crypts as seen by increased fission and then a return to homeostatic levels [23]. In these examples as in our data, crypt fission appears to reflect ISC expansion and allows for increasing numbers of crypts in a process that is closely regulated during development and after ileocecal resection.

Cheng and Bjerknes have previously quantified the overall proliferative activity of murine intestinal crypts during postnatal development by flow cytometry and found proliferative indices between 7–14% [1]. They did not identify changes in proliferation temporally corresponding to the surge in crypt fission. In fact, there was a drop in the percentage of cells in S phase with a nadir on p21, when the rate of fission was still markedly elevated in their mice and significantly elevated in ours.

We identified distinctive patterns of mRNA expression among several putative ISC markers as shown in Fig. 3. The first marker to show elevated expression was *mTert*, at p4. This finding was followed by surges in *Lgr5* and *Bmi1*, which peaked at p14, corresponding with the peak of crypt fission. Interestingly, the early increase in *mTert* mRNA correlates with recent data indicating that putative ISCs marked by *mTert* may give rise to *Lgr5* ^+^ cells [17]. In contrast, *Olfm4* mRNA levels were significantly lower at all timepoints in development compared with adult while *Ascl2* was statistically similar to adulthood at p7–14 during the time of elevated crypt fission and lower than adult levels at the other timepoints (Table S1). As mentioned, *Lgr5*, *Olfm4*, and *Ascl2* are thought to represent the same group of ISCs, marking the crypt base columnar ISC sub-population [15], [20], [21]. However, in our study the latter 2 markers were expressed differently from *Lgr5* and their expression did not parallel crypt fission levels (Fig. 2A and Table S1). This observation of divergent mRNA expression among markers of ISCs has been noted before with *Olfm4* [29]. Such divergent behavior stands as a caution that relationships between markers established in adult animals under homeostatic conditions do not necessarily hold during development or during other physiological or pharmacological challenges.

Prior work utilizing SP sorting has demonstrated it to be a population enriched for putative ISCs [25]. Hypothesizing that this technique would be similarly useful in the developmental period, we quantitated the SP fraction from p21 mice as compared with adult. The results (Fig. 3F) showed the% SP at p21 to be 8-fold higher than in adulthood (4.8 vs. 0.58). This correlates closely with crypt fission, which showed a 6-fold difference between p21 and adult (Fig. 2A). Although p21 was not the peak of crypt fission, given the fragile nature of neonatal intestinal tissue, we utilized the latest time point at which crypt fission was significantly elevated compared with adult animals in order to obtain the best epithelial preparation possible. This provided additional evidence that there was expansion of ISCs at the time of elevated crypt fission in our model. As cell cycle analysis of hematopoietic SP has shown this fraction to represent cells in G0 [30,31], our demonstration of elevated % SP may be linked with the behavior of the quiescent ISC population.

In tissues with complex cellularity such as the intestine, rising levels of mRNAs marking a subset of cells are often taken to indicate an increase in the number of cells producing that particular mRNA. Alternatively, it may reflect an increase in the amount of mRNA produced per cell if the number of cells remains constant. In order to analyze the relationship between ISC mRNA markers and ISC numbers in the developing intestine we would ideally use antibodies to markers of the two putative ISC populations in order to quantitate their numbers. Unfortunately at this time such antibodies are not available. Thus we used the commercially available *Lgr5*-EGFP mouse.

Taking this approach to assess ISC numbers in the developmental period allowed for visualization of *Lgr5*-expressing cells via fluorescence. The increased expression of *Lgr5* mRNA (Fig. 3A) was not seen as an increased percentage of EGFP positive cells per crypt. Interestingly, the percentage of total crypts that were EGFP positive was significantly lower in adulthood compared with all developmental timepoints and highlights the varying degree of mosaic EGFP expression present in this animal. Within the limitations of this particular animal model, the fact that EGFP positive cells were extremely rare at birth may be significant. Given their rarity, we elected not to include that timepoint in statistical analysis. However, with that caveat, there is certainly an obvious increase in EGFP expression at the forming crypt base from p0 to p7 (Fig. 4A & B). This observation is supported by the original paper describing *Lgr5*, which reported minimal *Lgr5* expression around the time of birth [15].

*In situ* hybridization of *Lgr5* mRNA in WT mice confirmed the lack of expression at birth (Figure S1). At 1 week, expression of *Lgr5* occurred in all crypts, to support the mosaic nature of the *Lgr5*-EGFP mouse, rather than variable expression of *Lgr5* itself. Over time, the autoradiographic signal remained strong in the crypts. Although not quantifiable, the number of *Lgr5*-expressing cells in the crypts appears to remain relatively constant from p7 into adulthood, which is similar to our EGFP data as shown in Fig. 4. Overall, our data with the *Lgr5*-EGFP mouse and *Lgr5 in situ* hybridization suggest that *Lgr5*-expressing ISC may be involved in the initial formation of crypts during the first postnatal week and then remain relatively constant in number, consistent with their proposed role in steady state turnover of the intestinal epithelium.

Different mouse models offer a variety of circumstances to investigate the process of crypt generation as it relates to ISC biology. Following ileocecal resection, increases of ISC numbers (measured by SP sorting, phospho-β-catenin staining and bromodeoxyuridine label retention) have been reported to precede the increase in crypt fission [23]. Interestingly, all of the ISC parameters utilized in that study tend to characterize the “plus 4” quiescent subpopulation of ISCs. To date, specific assessment of the jejunal CBC subpopulation following ICR has not been reported, however other investigators demonstrated no significant increase in ileal *Lgr5* expression following 50% small bowel resection [32]. In animals treated with doxorubicin, significant surges in crypt fission and% SP are seen with regeneration of crypts starting 1 week post-injury, but there is no increase in either the number of *Lgr5*-expressing cells or *Lgr5* mRNA levels at similar time points [26].

Clearly, techniques to directly evaluate actual numbers of ISCs are lacking. With this limitation, surrogates are used to indirectly study ISC expansion. In development, elevated levels of crypt fission correspond with rapid increases in small bowel length, circumference, and the number of crypts per circumference over a discrete time period. We describe patterns of mRNA expression in this time frame for several putative ISC markers and show that *Lgr5*, *Bmi1*, and *mTert* are elevated during and/or preceding the surge in crypt fission while *Olfm4* and *Ascl2* are not. Crypt fission is a unifying theme in 3 distinct scenarios that involve expansion and/or repair of the intestinal epithelium. Neonatal development is strictly a growth phenomenon while the adaptive process after ileocecal resection is a response of normal bowel to a loss of length elsewhere in the gastrointestinal tract. The response to damage arises from crypts that have sustained an insult whether from radiation or chemotherapeutic agents.

We provide evidence that during development, a heterogeneous population of ISCs, both active (as seen via *Lgr5* mRNA expression, *Lgr5*-EGFP, and *Lgr5 in situ* hybridization) and quiescent (as seen by mTert mRNA, Bmi1 mRNA and% SP), may play a role in neonatal intestinal growth. While the surge of crypt fission seen at the end of the second postnatal week suggests increased numbers of ISC at this time, we were not able to demonstrate a clear correlation between this surge and either of these populations. This raises the question that both active and quiescent ISC subpopulations may be interacting in a complex manner during neonatal intestinal growth. Further characterization is difficult within the current limitations of the field, namely the lack of any specific, commercially available antibodies for putative ISC markers. All of the measures currently available are surrogates. A better understanding of the regulatory mechanisms controlling crypt fission and the expansion of ISCs has clear translational application to patients suffering from short bowel syndrome and other intestinal pathology. Enhanced techniques to more directly identify, quantify, and manipulate putative ISCs have significant potential. Further characterization of ISC biology may reveal components that are subject to beneficial clinical intervention.

## Supporting Information

Figure S1 *Lgr5 in situ* hybridization autoradiographs using ^35^S-labeled antisense probes are shown at low power (panel A) and high power (panel B).
(TIF)

Figure S2 Anti-sense and sense controls for *Lgr5 in situ* hybridization.
(TIF)

Table S1 Statistical analysis of mRNA expression and crypt fission. P-values indicate comparison with adult values for each mRNA and for crypt fission.
(TIF)
