# Supplementary figures and images for: Expansion of Intestinal Epithelial Stem Cells during Murine Development
Source: PLoS One. 2011 Nov 10;6(11):e27070. doi: 10.1371/journal.pone.0027070 (PMC3213109; doi:10.1371/journal.pone.0027070)

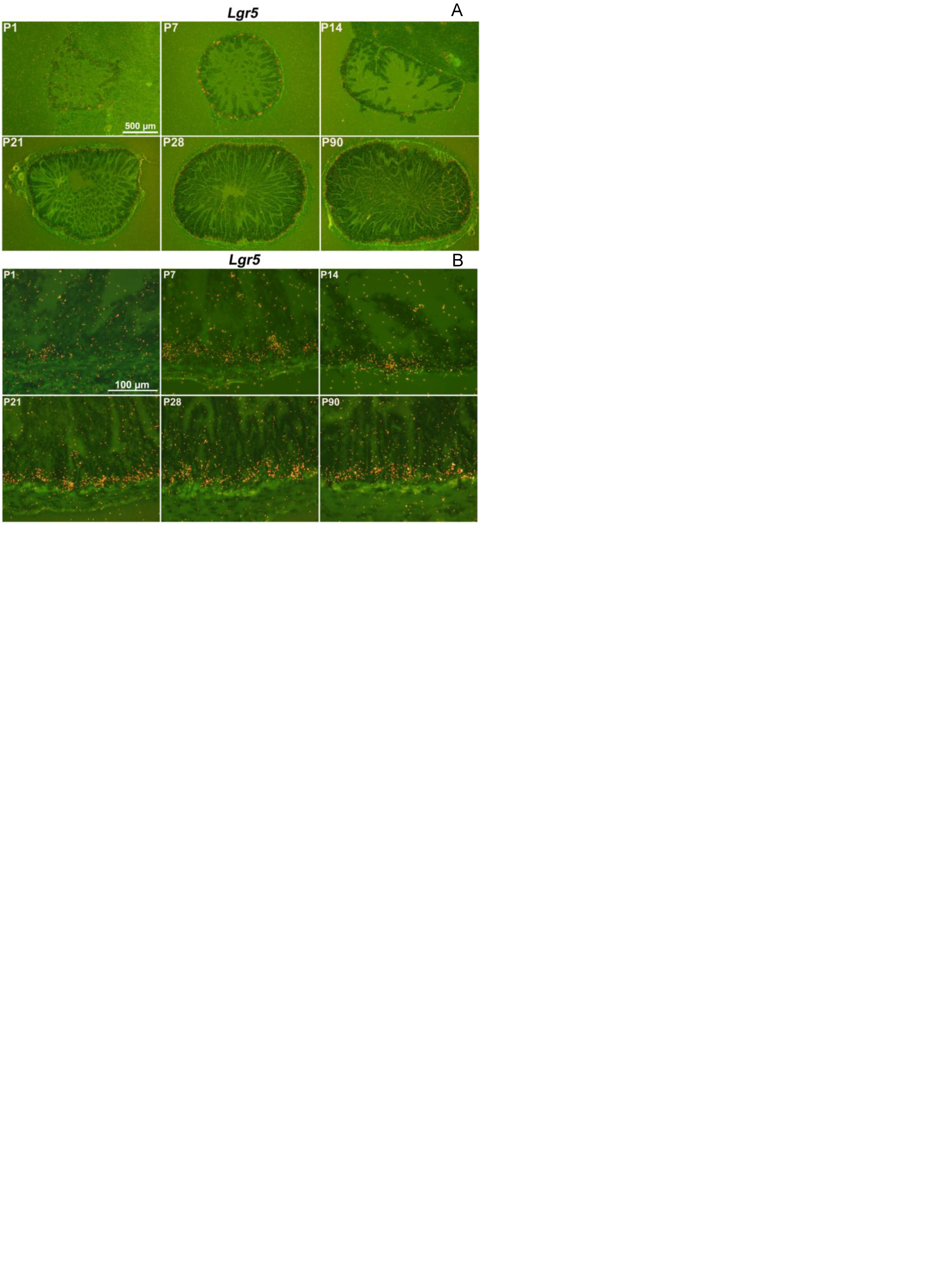

Supplement: Figure S1 — Lgr5 in situ hybridization autoradiographs using 35S-labeled antisense probes are shown at low power (panel A) and high power (panel B). (TIF) [file pone.0027070.s001.tif]

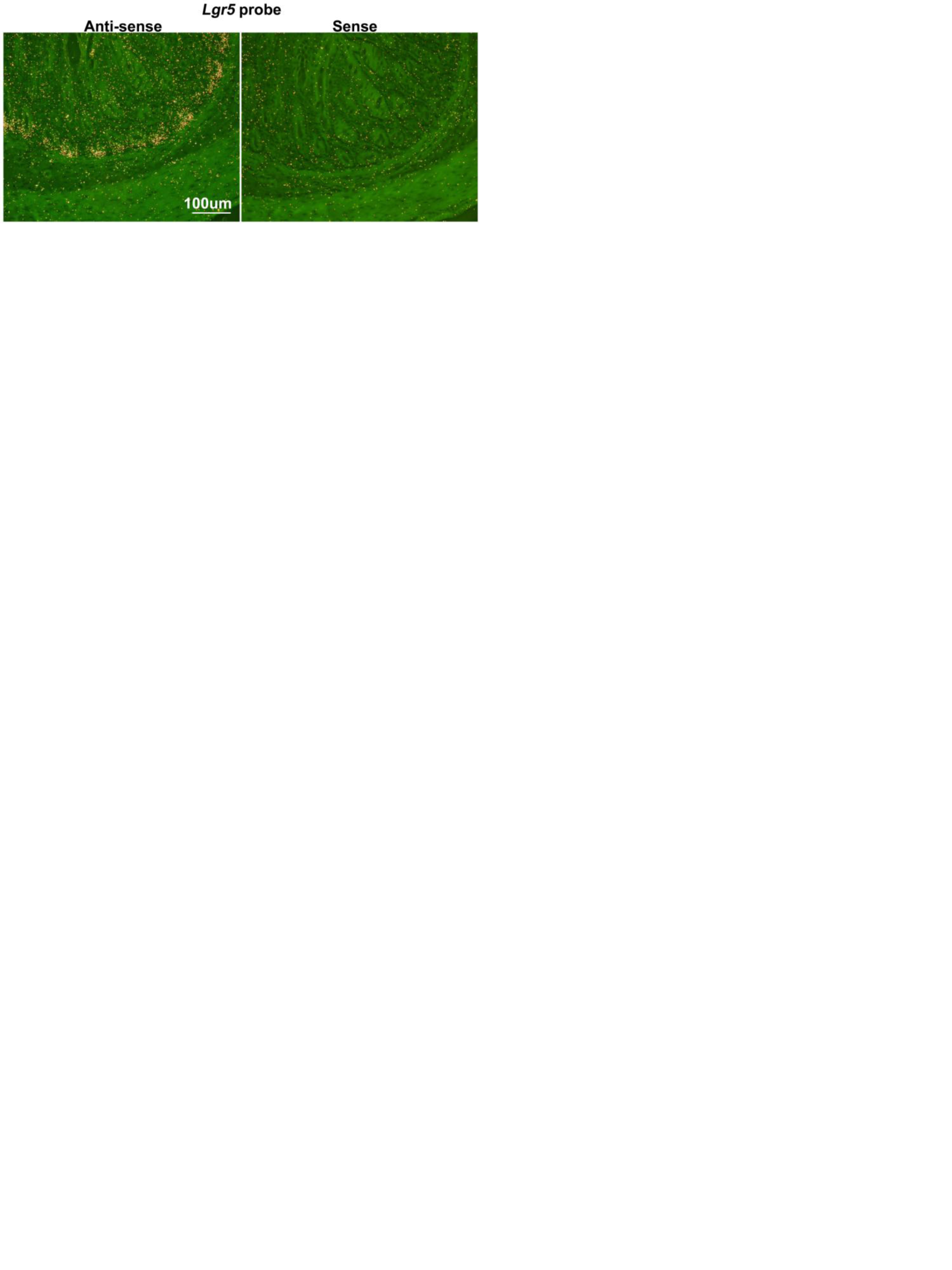

Supplement: Figure S2 — Anti-sense and sense controls for Lgr5 in situ hybridization. (TIF) [file pone.0027070.s002.tif]

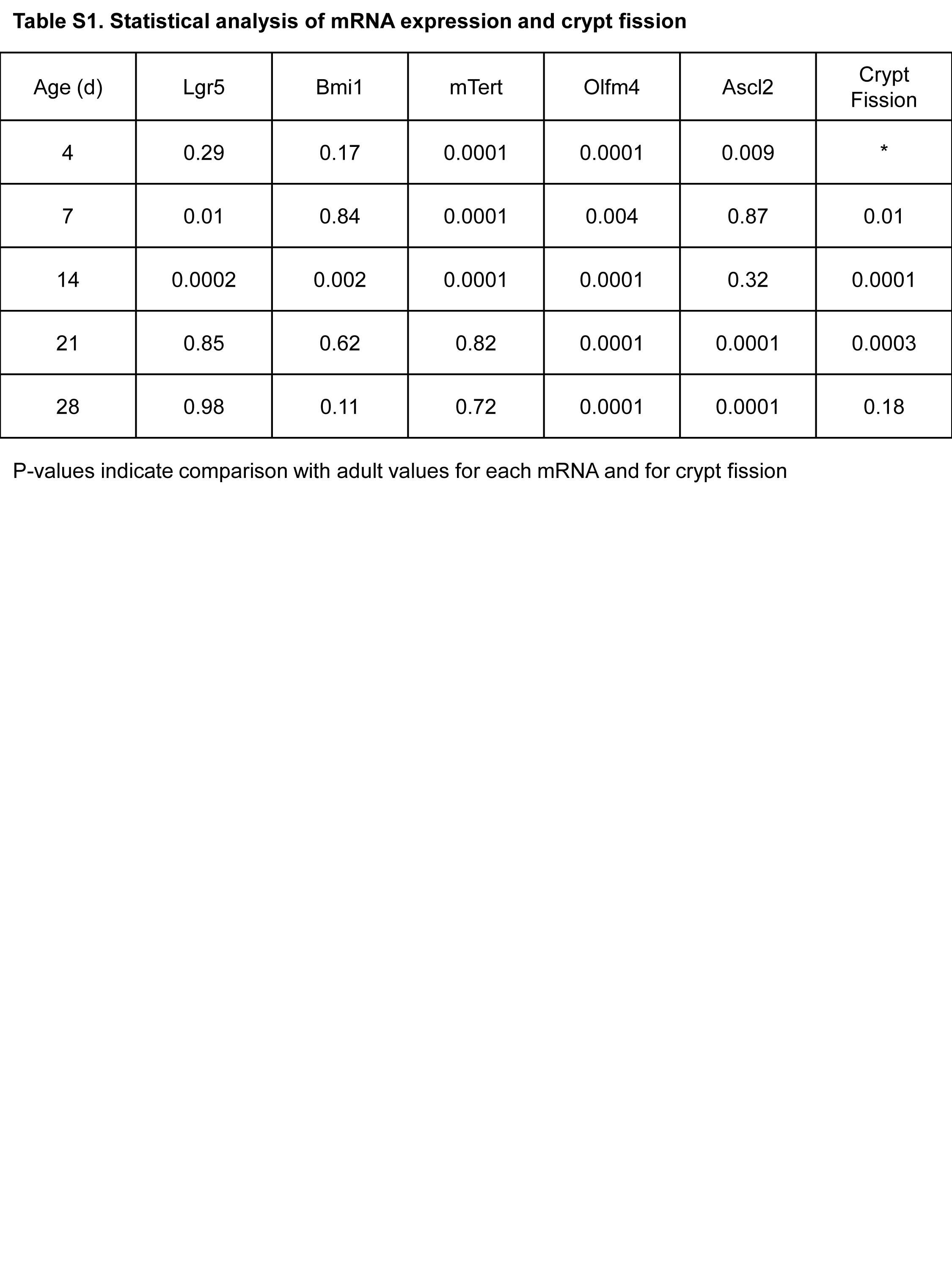

Supplement: Table S1 — Statistical analysis of mRNA expression and crypt fission. P-values indicate comparison with adult values for each mRNA and for crypt fission. (TIF) [file pone.0027070.s003.tif]
